# Circadian biology and exercise: the time to move

**DOI:** 10.1093/lifemeta/loag009

**Published:** 2026-03-28

**Authors:** Zhihui Zhang, John A Hawley, Min-Dian Li

**Affiliations:** Department of Cardiovascular Medicine, Southwest Hospital, Army Medical University, Chongqing 400038, China; Key Laboratory of Chronobiology and Cardiometabolic Disease, Chongqing Education Commission of China, Chongqing 400038, China; Key Laboratory of Geriatric Cardiovascular and Cerebrovascular Disease (Army Medical University), Ministry of Education, Chongqing 400038, China; Centre for Human Metabolism and Performance, The Mary MacKillop Institute for Health Research, Melbourne, Victoria 3002, Australia; Department of Sport and Exercise Sciences, Manchester Metropolitan University Institute of Sport, Manchester M15 6GX, United Kingdom; Department of Cardiovascular Medicine, Southwest Hospital, Army Medical University, Chongqing 400038, China; Key Laboratory of Chronobiology and Cardiometabolic Disease, Chongqing Education Commission of China, Chongqing 400038, China; Key Laboratory of Geriatric Cardiovascular and Cerebrovascular Disease (Army Medical University), Ministry of Education, Chongqing 400038, China

**Keywords:** metabolic health, circadian clock, circadian rhythm, exercise, exercise training, skeletal muscle

## Abstract

Exercise performance in endurance- and power-based events is time-of-day dependent in both humans and rodents. Accordingly, there has been growing interest in determining whether there is an optimal time of day for physical activity that can amplify the well-known benefits of exercise on metabolic health in humans. Here, we discuss critical features of circadian biology that underpin many of the physiological responses to the timing of exercise. Recent studies indicate that the circadian clock regulates exercise capacity through the coordination of tissue-specific physiological responses, including fuel metabolism and mitochondrial biogenesis. Synchronized actions between circadian clocks and clock-output pathways residing in the skeletal muscle and other tissues are likely to explain how external time-of-day cues influence exercise performance and physiological responses to exercise. Understanding the circadian biology of exercise will provide the foundation on which future individualized exercise protocols are prescribed to improve metabolic health outcomes at both individual and population levels.

## Introduction

Exercise is medicine [1]. It makes the weak strong and the strong well, and enhances whole-body metabolic health [2, 3]. The molecular mechanisms underpinning the physiological responses and adaptation to exercise have been extensively studied over the past two decades, with the application of molecular techniques to exercise biology offering a greater understanding of the multiplicity and complexity of cellular networks involved in exercise responses [4–6]. Recent discoveries have also highlighted the putative mechanisms by which the skeletal muscle communicates with other organs and mediates many beneficial effects of exercise on health and performance [7–10]. However, only during the past decade has there been growing recognition that the time of day is an important component of exercise biology, and synchronizing physical activity and/or meals to a specific time of day may amplify performance and the health benefits of exercise.

**Figure 1:**
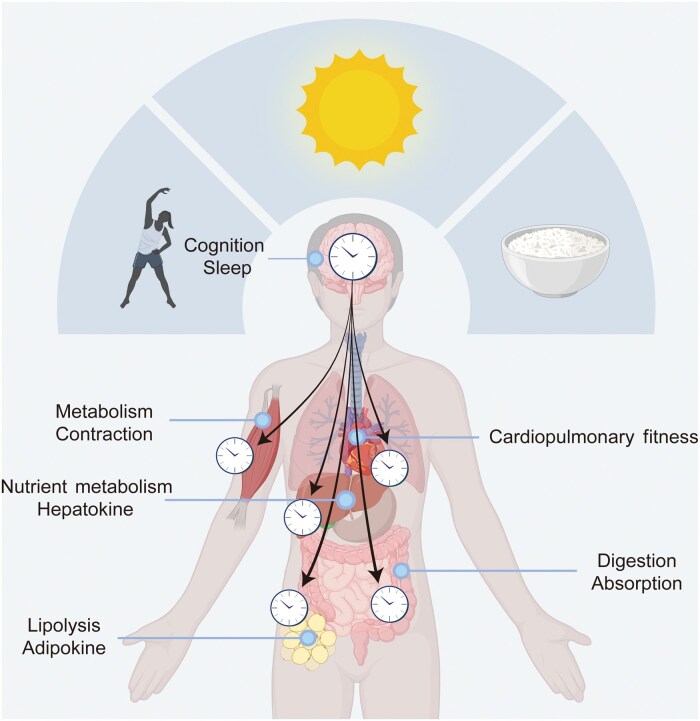
Time-of-day cues influence exercise biology. Daily cycles of day and night generate periodic changes in environmental cues and behavioral cues including, but not limited to, light/dark cycles, sleep/wake cycles, feeding/fasting cycles, and exercise/inactivity cycles. In physiological conditions, these time-of-day cues synchronize the circadian clock system in the brain and body, thus influencing the physiological and metabolic responses to exercise in both preclinical models and humans. Created with BioRender.com.

The 24-h biological clock (or circadian clock; “circa” meaning “about” and “dian” meaning “daily”) system and its alignment with external time-of-day cues are now recognized as important determinants of the physiological and metabolic responses to exercise in both preclinical (animal) models and humans [11–16] (Fig. 1). In this review we provide an overview of the circadian clock system, with a focus on the interaction between circadian biology and exercise, and discuss how the timing of exercise regulates the circadian clock system in the skeletal muscle, as well as in other tissues and organs. We review the mechanistic basis underpinning the impact of time-of-day on exercise biology and discuss how leveraging the circadian-exercise axis may help to improve metabolic health outcomes in humans.

## Overview of the circadian clock system

Daily cycles of day and night generate periodic changes in light (i.e., daily light/dark cycles), nutrient availability (i.e., daily feeding/fasting cycles, the timing and number of eating occasions each day), sleep/wake cycles, and daily timing of exercise (Fig. 1). These time-of-day cues synchronize the circadian clock system in the brain and peripheral tissues/organs, thereby influencing many biological processes [17–19].

### Central and peripheral clocks

The circadian clock system is organized in a hierarchical manner and can be conceptualized as consisting of the central clock and peripheral clocks [20–24]. The central clock resides in the suprachiasmatic nucleus (SCN) in the hypothalamus and gene­rates self-sustaining rhythms lasting approximately 24 h, affecting behavior and synchronizing neuroendocrine hormones to exert their action on target tissues [20, 21]. The central clock (or the SCN clock) is calibrated by light/dark cycles and coordinates circadian clocks in peripheral tissues, including the liver, skeletal muscle, and adipose tissues, via rhythmic behaviors, neural and humoral cues [25]. The structure between the SCN clock and extra-SCN clocks (known as peripheral clocks) is thus hierarchical [20, 21, 23, 24, 26, 27]. Nutrient availability is a potent time cue for peripheral clocks in the liver and visceral adipose tissue, whereas the timing of exercise can reset skeletal muscle clocks [28–31].

**Figure 2:**
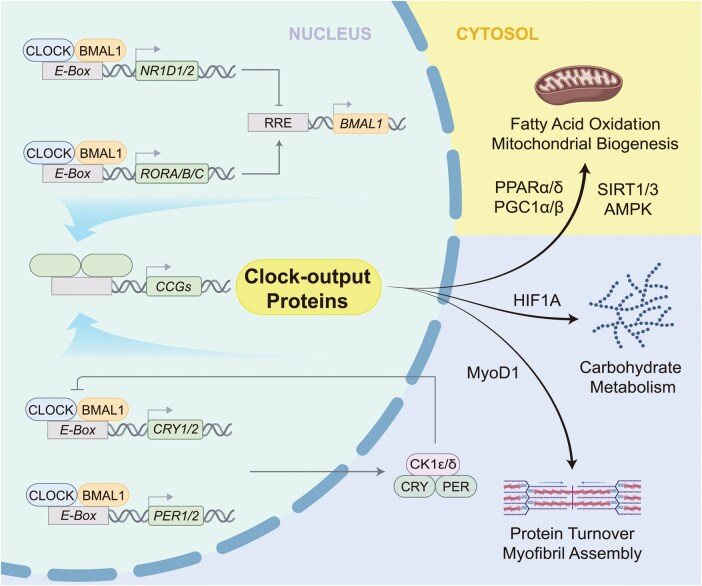
Molecular mechanisms of the circadian clock in myocytes. Transcription factors BMAL1 and CLOCK activate the transcription of Period (*PER1* and *PER2*) and Cryptochrome (*CRY1* and *CRY2*) genes. PER and CRY assemble into a protein complex that represses the expression of their own genes by binding to BMAL1 and CLOCK, thus closing the core feedback loop. These and several additional interlocked transcriptional−translational feedback loops (e.g., REV-ERBs, encoded by *NR1D1* and *NR1D2*, and RORs, encoded by *RORA*, *RORB*, and *RORC*) form the circadian clock at the cellular level. The core clock in myocytes (the muscle clock) coordinates daily rhythms in FA oxidation and mitochondrial biogenesis before wake time, as well as carbohydrate metabolism and muscle growth in the active phase via selected clock-controlled genes (CCGs) that encode clock-output proteins. AMPK, AMP-activated protein kinase; BMAL1, brain and muscle ARNT-like protein 1; CK1, casein kinase 1; CLOCK, circadian locomotor output cycle kaput protein; HIF1A, hypoxia-inducible factor 1 subunit alpha; MyoD1, myogenic differentiation 1; PPAR, peroxisome proliferator-activated receptor; PGC1, PPARγ coactivator 1; RRE, ROR response element; SIRT, sirtuin. Created with BioRender.com.

Both central and peripheral clocks share a set of circadian clock genes, which form the core of a circadian oscillator in almost all cell types and tissues (hereafter referred to as the core clock) [24, 32]. In mammalian cells, transcription factors including brain and muscle ARNT-like 1 (BMAL1) and circadian locomotor output cycles kaput (CLOCK) activate the transcription of *Period (Per1* and *Per2)* and *Cryptochrome (Cry1* and *Cry2)* genes [17]. PER and CRY assemble into a protein complex that represses the expression of their own genes by binding to BMAL1 and CLOCK, thus closing the core feedback loop. To stabilize the core feedback loop, accessory feedback loops exist. For example, nuclear receptors REV-ERBα/β (reverse c-erbA α/β, encoded by *Nr1d1* and *Nr1d2*) and RORα/β/γ (retinoic acid-related orphan receptors, encoded by *Rora*, *Rorb*, and *Rorc*) are rhythmically trans-activated by BMAL1 and CLOCK, protein products of which repress and activate the expression of the *Bmal1* gene, respectively. These and several additional interlocked transcriptional-translational feedback loops form the circadian clock at the cellular level (Fig. 2). Comprehensive discussion of the mechanisms underpinning the regulation of circadian clocks can be found elsewhere [17, 33–37].

### Clock outputs to muscle physiology

While the core clocks are similar between tissues, the clock-regulated circadian biological processes are tissue-specific [38–41]. Genome-wide transcriptome studies report that 40%−80% of all protein-coding genes exhibit circadian rhythm in at least one tissue [38–40], while proteome-wide studies reveal that the levels of proteins and post-translational modi­fications are also rhythmic in central and peripheral tissues [42–49]. It is estimated that rhythmic transcripts rarely overlap between tissues except for those related to the circadian clock [38, 40]. This raises the notion that the core clock synergizes with tissue-specific clock-output proteins and communicates circadian rhythms to numerous tissue-specific biological processes [13, 31, 50–54]. In such a paradigm, clock-output proteins (i.e., products of selected clock-controlled genes) are regulated by both the circadian clock and cellular signaling, and transmit rhythmicity to tissue-specific biological processes. Conceptually, these clock-output proteins could help explain the remarkable tissue specificity of circadian transcriptomes. Phenotyping studies of mice with mutations in core clock genes (e.g., *Bmal1* and *Clock*) link physiological functions to circadian regulation, but there is always a concern that circadian clock proteins are transcriptional factors that have plausible non-clock functions. As such, the function of a tissue clock is validated based on the comparison between genetic models of clock-controlled genes and those of core clock genes.

In the skeletal muscle, the muscle clock regulates key biological processes related to metabolism and growth, including fatty acid (FA) oxidation, mitochondrial biogenesis, carbohydrate (CHO) metabolism, protein turnover, and myofibril assembly [13, 55] (Fig. 2). A well-established muscle-specific clock-output protein is myogenic differentiation 1 (MyoD1, encoded by *Myod1*), a master regulator that facilitates circadian control of muscle growth and contraction [56–59]. MyoD1 amplifies circadian signaling derived from the muscle clock into myofibril assembly and dynamics [56]. As demonstrated in genetic knockout mice, depletion of *Myod1* causes defects in the skeletal muscle structure related to the muscle clock but not those related to mitochondrial functions [58].

CRY1/2 rhythmically bind to and repress clock-output proteins, including peroxisome proliferator-activated receptor delta (PPARδ) and hypoxia-inducible factor 1 alpha (HIF1A), for the control of mitochondrial energy metabolism and hypoxia-induced responses in the skeletal muscle, essential for submaximal exercise tasks [60, 61]. While REV-ERBα and REV-ERBβ are functionally redundant in maintaining the circadian clock and metabolism in other tissues [62, 63], REV-ERBα promotes FA oxidation and mitochondrial biogenesis, whereas REV-ERBβ inhibits these processes in the skeletal muscle [64–66]. The 24-h sequential activity of REV-ERBα and REV-ERBβ ensures daily patterns of lipolysis and FA oxidation before activity/night onset in the skeletal muscle in mice [64–66]. Nutrient sensors, such as the PPARγ coactivator 1 alpha/beta (PGC1α/β), sirtuins (SIRT1/3), AMP-activated protein kinase (AMPK), and HIF1A, are engaged by the circadian clock to regulate mitochondrial functions, autophagy, and CHO metabolism (Fig. 2; described subsequently). Within the skeletal muscle resides a local stem cell population, muscle stem cells (MuSCs), also known as satellite cells, which has their own clock. The clocks in myofibers and MuSCs coordinate the circadian timing of muscle regeneration [67–71].

## Circadian components of exercise biology

Exercise capacity is determined by the complex interaction of intrinsic and extrinsic factors [4], with issues relating to the speed, force, duration, and intensity of muscle contractions that limit performance [3, 4]. In both humans and mice, the physiological and metabolic responses to exercise are time-of-day dependent, leading to substantial variation in exercise performance [6, 12]. For example, circadian variations in peak performance are found in endurance- and power/speed-based activities in rodents [72, 73], healthy but untrained individuals [72], and elite athletes alike [74, 75]. While daily cycles of body temperature and hormones are key mediators of the time-of-day effects on exercise performance, accumulating evidence indicates that the circadian clock system may be an essential component of exercise biology [11–13, 76]. Here, we highlight how circadian cues involving the core clock mediate the responses to exercise.

### Exercise performance

**Figure 3:**
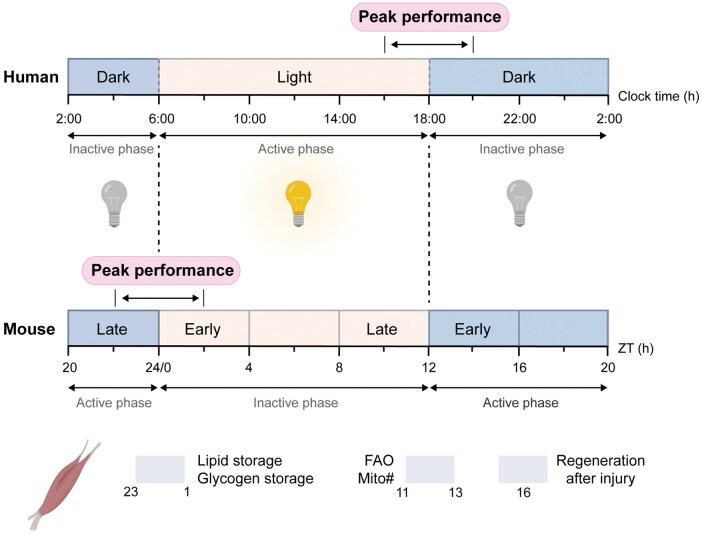
Summary of circadian timing related to peak performance and physiological responses to exercise in humans and mice. Exercise refers to endurance- and power/speed-based physical activities. ZT, zeitgeber time; FAO, fatty acid oxidation; Mito#, mitochondrial content. Created with BioRender.com.

Exercise power output/speed and endurance capacity typically exhibit peak performance in the late afternoon/evening at around 16:00–20:00 in humans (Fig. 3), with an effect size ranging from 1.7% to 14% depending on the training/performance status of the individual and the modality of exercise [11, 72, 74, 77–79]. Such an effect size is less in elite athletes [74, 80, 81]. Despite well-planned and structured schedules, including morning and evening training sessions, time-of-day effects exceed the difference between medalists and finalists among Olympic swimmers [81]. The times of day for peak performance in athletes are different between “larks” and “owls” (i.e., morningness/eveningness circadian phenotype), with circadian phenotype and the time since entrained awakening (i.e., entrainment status of the circadian system reflecting internal biological time) being the major predictors of peak performance times [74]. As such, physical performance in humans is not only determined by the time of day of training, but also by the circadian clock system.

Given their nocturnal propensity for physical activity, mice exhibit peak performance in the biological evening at around zeitgeber time 22–2 h (ZT0 denotes zeitgeber time 0 h or the time when the lights are turned on in a laboratory facility that follows a 12-h light and 12-h dark schedule), as demonstrated by their enhanced running performance [15, 72, 73, 82, 83] (Fig. 3). This time-of-day exercise effect is mechanistically controlled by the circadian clock, as disruption of the circadian clock, such as in *Per1/2* double knockout or *Bmal1* knockout mice, abolishes the time-of-day differences in exercise power output and endurance [15, 72]. Such time-of-day differences persist under constant darkness, ruling out the confounding effect from light/dark cycles [15]. Genetic analysis has revealed that the *Per2* gene is sufficient for circadian regulation of exercise performance [15], although myocyte *Per2* is dispensable [49, 84]. The time-of-day effect of the circadian clock can be compensated for by scheduling physical training within the active phase [15, 82, 83] (Fig. 3). Taken collectively, the results from these studies highlight the strong association between exercise performance and circadian clocks.

### Fuel metabolism

In humans, CHO-based fuels (i.e., muscle and liver glycogen, blood glucose, and blood lactate) are the predominant sources of energy for the skeletal muscles during sustained, continuous exercise lasting several minutes or longer, with the relative contribution of these metabolic pathways primarily determined by the intensity and duration of exercise [85–87]. Mice have a greater reliance on extracellular substrates from metabolic tissues (i.e., blood glucose, free FAs [FFAs], and lactate) [88–90].

**Figure 4:**
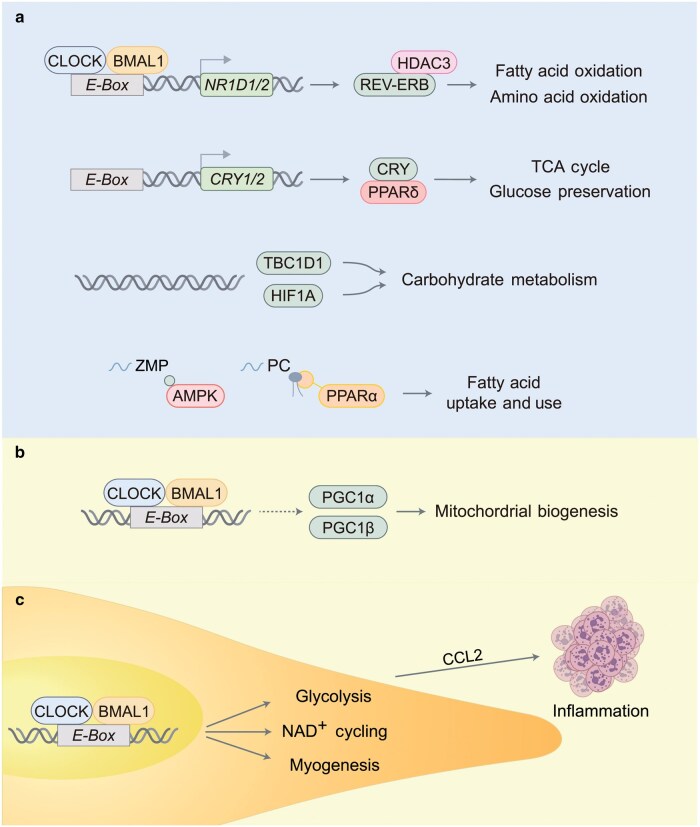
Circadian components of exercise biology. (a) Fuel metabolism in the skeletal muscle is governed by the muscle clock and nutrient sensors. (b) Mitochondrial biogenesis in skeletal muscle is regulated by rhythmic expression and activity of PGC-1α/β. (c) Muscle regeneration after injury is orchestrated by the MuSC clock.

The muscle clock plays a major role in substrate selection to fuel physical activity in mice (Fig. 4a). BMAL1 regulates insulin sensiti­vity and CHO metabolism in muscle tissues, in part via circadian expression of key genes involved in glucose uptake (*Tbc1d1*) and the citric acid cycle (*Pdp1*) [55, 58, 91–93]. Muscle-specific *Bmal1* knockout induces gene expression patterns to favor lipid storage and metabolism, including perilipin 5 (*Plin5*, lipid droplet biogenesis) and the solute carrier family 27 member 1 (*Slc27a1*, FA oxidation), at the expense of CHO utilization, and increases the expression of lipogenic genes in a time-of-day manner [94]. In line with these findings, adeno-associated vector-mediated rescue of muscle Bmal1 expression in *Bmal1* knockout mice improves systemic glucose tolerance, muscle strength, and metabolism [91]. In contrast, double knockout of *Cry1/2* genes increases muscle glycogen storage, PPARδ-mediated exercise responses, and sprint exercise performance in mice [60]. PPARδ activates a subset of the genetic program for utilization of FAs and amino acids, thus sparing blood glucose during exercise [95]. The alpha isoform of PPAR (PPARα) is activated by phosphatidylcholine (PC)18:0/18:1 for the daily rhythm of FA uptake and oxidation in the skeletal muscle [96]. The circadian repressor REV-ERBα promotes FA oxidation in the skeletal muscle and enhances exercise capacity [65], while REV-ERBβ inhibits FA oxidation and subsequently blunts exercise performance [66]. These observations are consistent with the findings that muscle REV-ERBα/β recruit nuclear receptor corepressor 1 (NCoR1) and histone deacetylase 3 (HDAC3) for rhythmic outputs, and skeletal muscle-specific depletion of either NCoR1 or HDAC3 markedly enhances exercise endurance [97–99].

### Mitochondrial biogenesis

The metabolic demands of exercise require robust mitochondrial energy metabolism to ensure a timely continuous supply of ATP for muscle contraction [4, 85]. However, energy metabolism in the mitochondria is associated with the production of reactive oxygen species (ROS), with excessive ROS production leading to oxidative stress and compromised cellular physiology. As physical activity exhibits a 24-h cycle, the mitochondria can adapt to the time-of-day dependent need of bioenergetics by circadian control of mitochondrial number and dynamics. Indeed, the muscle clock coordinates circadian rhythms of mitochondrial biogenesis and various processes, such as mitochondrial fusion, fission, and mitophagy [13].

The circadian clock regulates mitochondrial biogenesis in the skeletal muscle (Fig. 4b). Muscle mitochondrial number exhibits peak abundance in the biological morning at around ZT11–13 under a circadian-aligned feeding schedule in mice [49]. Disruption of the muscle clock reduces the mitochondrial volume and density by up to 40%, and impairs mitochondrial respiration and coupling in the skeletal muscle [58]. The circadian clock regulates *PGC1α* expression and coordinates circadian pattern of *PGC1β* transcripts in the skeletal muscle [100]. PGC1β promotes voluntary physical activity particularly in the active phase [101] (see Fig. 3). The circadian repressor REV-ERBα regulates mitochondrial biogenesis via transcriptional control of the AMPK−SIRT1−PGC1α axis and autophagy in the skeletal muscle [65]. The circadian clock is involved in mitochondrial ROS production and detoxification through the control of NAD^+^ metabolism, antioxidant defense, and mitochondrial dynamics [102, 103]. This regulation involves a direct transcriptional control by circadian regulators, such as BMAL1-CLOCK and SIRT1/3-dependent post-translational modifications [103–108].

### Muscle regeneration after injury

Muscle repair and regeneration after exercise-induced damage are under the control of circadian clocks [109] (Fig. 4c). Recent studies have demonstrated that muscle injury activates an hypoxic response and recruits MuSCs and immune cells for tissue regeneration in a time-of-day manner [67–71]. Circadian clock proteins regulate the hypoxic response via hypoxia-inducible factors (HIFs) in many organs, including the skeletal muscle [110–115]. The circadian clock gates diurnal activity of HIF1A signaling and glycolysis in the skeletal muscle, with the maximal hypoxic response to strenuous exercise occurring in the early active phase at ZT16 [110] (see Fig. 3). MuSCs are activated from a quiescent state after muscle injury to initiate myogenic differentiation [116]. The muscle repair capacity is higher at ZT16 in mice, and loss of *Bmal1* in MuSCs reduces the rates of glycolysis and NAD^+^ metabolism, impairing myofiber differentiation [69, 70]. Activated MuSCs mobilize oxygen-independent glycolysis and NAD^+^ to induce the expression of the chemotactic C–C motif chemokine ligand 2 (CCL2) for more efficient recruitment of neutrophils in the regenerating muscle niche at ZT16, which is controlled by the MuSC clock [71].

MuSCs gate time-of-day differences in *ex vivo* contractility and mitochondrial respiration [68, 77]. Following injury (i.e., eccentric overloading), the skeletal muscle undergoes repair via MuSC-mediated myogenic progression, with *Bmal1* being necessary for appropriate myogenic progression and repair [67]. Notably, the role of the MuSC clock in muscle regeneration is context-specific, and differs between exercise-induced contractile injury and chemical-induced injury. Details of circadian clock-controlled muscle regeneration after contractile injury require further investigation.

### Interaction of exercise with meal timing and nutrient availability

The entrainment by environmental/behavioral cues resets the circadian clock system and generates shifts in systemic and tissue metabolism [24, 34, 36, 50, 117]. Endurance exercise mobilizes energy substrates (predominantly CHO- and fat-based fuels) to support muscle contraction. Given numerous studies focusing on the muscle clock, the role of other peripheral clocks in exercise response and performance is an area where further research is needed. As substrate availability is critical in determining the response to exercise [4, 11, 12], recent studies on time-restricted eating (TRE), a paradigm that limits the daily window of nutrient intake to 8–12 h and plays off chronobiology [22, 31, 118–120], are of interest. A 9-h night/active phase-restricted feeding regimen in mice (NRF, akin to 8:16 TRE in humans) enhances endurance performance in both male and female mice during high-fat diet feeding [121], while an 8-h NRF improves motor coordination in high-fat diet-fed male mice [122].

Compared to NRF, day/inactive phase-restricted feeding (DRF) inverts the feeding schedule, resets the diurnal rhythmicity of peripheral clocks, and shifts tissue metabolism within several weeks in mice [42, 123–128]. DRF enhances endurance capacity without prior exercise training in both male and female mice, with this effect additive to physical training [49]. The muscle clock coordinates diurnal patterns of *PGC1α* and *Plin5* in the skeletal muscle under DRF [48, 49]. Of note, the circadian pace governing muscle mitochondrial metabolism is in synchrony with peripheral tissues, such as visceral adipose tissue, which is under the control of adipocyte AMPK signaling [48]. Adipocyte AMPK gates the diurnal rhythmicity of a plethora of metabolites in the blood, including lactate, succinate, FFAs, and uridine, which may mediate part of the fat−muscle tissue crosstalk [48]. Circadian-timed activation of adipocyte AMPK using chemical Compound 29 enhances exercise performance and the muscle clock in mice [48]. These studies provide evidence that coordinated meal timing can augment exercise performance partly through the circadian clock system, and that the exercise–nutrient interaction will be an important target in future efforts directed towards precision interventions to amplify the effects of exercise on metabolic health.

## Physiological adaptations to ­exercise timing

Depending on an individual’s fitness level and the prevailing intensity of the task, a single bout of voluntary dynamic exercise represents a major challenge to whole-body homeostasis, largely due to the increased metabolic activity of contracting skeletal muscles [4]. Multiple integrated and often redundant responses operate to blunt the homeostatic threats generated by exercise-induced increases in muscle energy and oxygen demand [4]. The molecular basis for skeletal muscle adaptation to repeated exercise challenges (i.e., exercise training) is characterized by marked cellular reprogramming of metabolic pathways through the activation of mechanical and metabolic sensors. This adaptation fundamentally involves increased expression and activity of key proteins, which is mediated by an array of signaling events, pre- and post-transcriptional processes, protein synthesis, post-translational regulation, and modulation of protein (enzyme) activities and/or intracellular localization [4, 129].

For humans, the most favorable time of day to undertake physical activity to amplify the health benefits of exercise is currently not well defined and depends on many factors, including the moda­lity and intensity of exercise, the entrained wake–sleep cycle, the timing of meals, an individual’s circadian phenotype, and the health status of the individual. However, emerging evidence from preclinical studies supports the notion that the circadian clock system, including the skeletal muscle clocks, is involved in the time-of-day responses to exercise. In the following sections, we discuss several tissue-specific physiological responses to exercise undertaken at different times of the day, specifically, the timing of endurance-based exercise and the related health benefits.

### Bone

**Figure 5:**
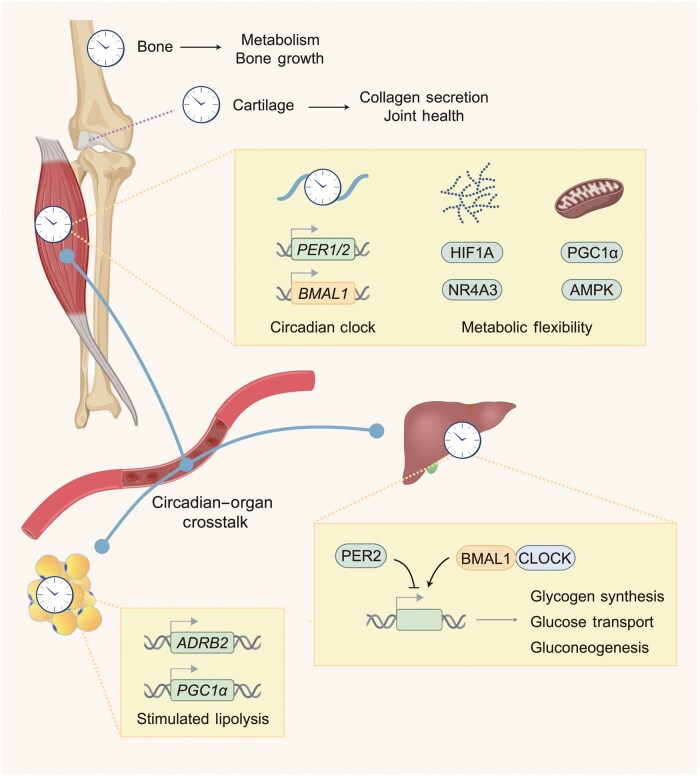
Circadian physiological adaptations to exercise timing. Endurance exercise at the early active phase (ZT12–16 for mice) induces maximum responses in the musculoskeletal and metabolism systems in lean mice. The bone clock promotes metabolism and bone remodeling for bone growth. The cartilage clock promotes collagen secretion and joint health. The skeletal muscle adapts through alterations in the circadian clock and metabolic flexibility. The liver clock adapts by coordinated regulation of circadian carbohydrate metabolism such as glycogen synthesis, glucose transport, and gluconeogenesis. The inguinal adipose clock increases the capacity for stimulated lipolysis. Created with BioRender.com.

Exercise training leads to substantial remodeling of the skeletal system, including bone, cartilage, and bone marrow. In mice, exercise training (treadmill running) during the early active phase (see Fig. 3) promotes bone growth, which coincides with the peak endogenous activity of oxidative phosphorylation in the chondrification center [130]. The bone clock regulates bone remodeling and bone mass in part via diurnal rhythms of oxidative phosphorylation, glycerolipid metabolism, and FA metabolism [131]. The cartilage clock regulates diurnal synthesis and secretion of collagen to replenish the extracellular matrix and protects against age-related degeneration, such as osteoarthritis [132–134]. The cartilage clock is sensitive to mechanical load and osmolarity, and is finely synchronized to the daily rhythm of physical activity [135]. Of note, bone marrow resident adipocytes act as a key reservoir of lipids and hormones for the skeletal system [136], with adipocytes modulating circadian bone physiology via leptin-induced sympathetic tone [137]. While there is no evidence that bone and bone marrow metabolism are under circadian control, this is entirely plausible and would contribute in important ways to the circadian–exercise axis to influence whole-body physiology. Further work exploring these links is warranted. Currently, evidence suggests that endurance-based training undertaken by mice during the early active phase promotes bone growth and joint health by synchronizing peripheral clocks in these tissues (Fig. 5).

In humans, bone cells, such as osteoblasts and osteoclasts, have their own circadian clocks. Recent evidence using a constant routine protocol (where external factors, such as meal timing, physical activity, sleep/wake, and light/dark, are held constant) suggests that, in healthy men and women, the circadian clock regulates bone resorption by osteoclasts robustly. In contrast, circadian clock regulation of bone formation by osteoblasts is negligible [138]. As exercise synchronizes circadian muscle and bone clocks, it seems reasonable to suggest that circadian-aligned exercise training may mitigate some of the negative effects of inactivity and circadian disruption on bone loss, which is a significant factor predisposing to age-related conditions, such as osteoporosis and sarcopenia.

### Skeletal muscle

Exercise training promotes tissue-specific and whole-body adaptations that improve cardiorespiratory and metabolic fitness, enhance exercise capacity (strength and endurance), and confer numerous health benefits [4, 139]. The skeletal muscle adapts to exercise training by remodeling fiber types from glycolytic fibers to oxidative fibers, increasing mitochondrial content, biogenesis, and oxidative capacity, and increasing utilization of alternative fuels including lipids [140, 141]. In mice, the muscle clock coordinates physiological adaptation to exercise training and mediates up to half of the exercise-induced transcriptomic changes, including those related to energy metabolism and fuel utilization, in addition to master regulators, such as nuclear receptor NR4A3 and PGC1α [142, 143]. PGC1- and estrogen-related receptor (ERR)–induced regulator in muscle 1 (PERM1) is a mediator of both acute endurance exercise and exercise training, and promotes the skeletal muscle adaptation to exercise in part via activation of calcium-calmodulin (CaM)-dependent protein kinase II (CaMKII) and p38 MAPK and genes related to mitochondrial biogenesis [144–146]. The *Perm1* gene is rhythmically expressed in mouse skeletal muscle under DRF [49], suggesting that meal timing may pre-condition circadian physiological adaptation to exercise training via specific factors, including PERM1. Transcriptional factor EB (TFEB), a master regulator of lysosomal biogenesis, exhibits time-of-day activity of autophagy, including mitophagy, throughout the circadian cycle, together with other regulators of autophagy [147–150]. The skeletal muscle TFEB mediates the switch of fuel utilization from CHO to FAs during exercise, achieving this through exercise-induced translocation to the nucleus of myofibers [151].

Changes in energy demand and substrate flux during and after exercise activate nutrient-sensing pathways, in particular the AMPK pathway in the skeletal muscle [152]. AMPK is a protein kinase complex that acts as a regulator of cellular metabolism in response to changes in cellular bioenergetics and CHO availabi­lity [153, 154]. Muscle AMPK regulates a plethora of adaptations to exercise, including mitochondrial energy metabolism and nucleo­tide balance during exercise [155, 156], mitochondrial content, and contraction-stimulated glucose uptake after exercise training [157]. Folliculin-interacting protein 1 (FNIP1), an AMPK substrate, controls lysosomal and mitochondrial biogenesis [158]. Genetic knock-in studies have demonstrated that the skeletal muscle FNIP1 controls mitochondrial function and muscle fuel utilization during exercise via AMPK-mediated phosphorylation of Ser^220^ [156]. Given that phosphorylation of hepatic FNIP1 (Ser^220^) and muscle *Fnip1* mRNA exhibit diurnal rhythms in mice [42, 49], it is of interest to explore whether the skeletal muscle AMPK−FNIP1 axis serves as a key mediator of circadian physiological adaptation to exercise training.

The ability of AMPK to respond to metabolic cues and to directly modify circadian clock components corroborates its role as an important mediator of circadian metabolism [48, 159]. In support of this contention, ZMP (5-aminoimidazole-4-carboxamide ribonucleotide), an endogenous AMPK activator, is induced by exercise in a time-dependent manner to regulate key steps in glycolytic and FA oxidation pathways and enhance exercise capacity [72]. In addition, transcripts encoding AMPK subunits are induced upon endurance exercise in the skeletal muscle [160], and muscle AMPK mediates mitochondrial antioxidant response after exercise [161].

Exercise timing induces differential responses in the muscle clock. In mice, a single bout of endurance treadmill exercise is sufficient to delay or advance the phase of the muscle clock by approximately 1 h, depending on the time of day, an observation confirmed by *ex vivo* electrical pulse-induced contractile acti­vity [162]. This phase-entrainment effect recapitulates the phase response curve, a cardinal feature of circadian entrainment to photic cues [163]. Combining restricted access to wheel and food with skeleton photoperiod permits analysis of voluntary exercise timing throughout the 24-h cycle without introducing artificial stress responses [73, 164, 165]. Inactive phase exercise reduces the amplitude of core clock genes, including *Bmal1* and *Cry1* [73]. In contrast, voluntary exercise, which mostly occurs in the active phase, increases the amplitude of *Per2* expression among core clock genes in the skeletal muscle [83]. This circadian clock response to exercise timing is consistent with the observation that active phase exercise improves exercise performance in mice [82, 83].

Exercise timing induces differential responses in metabolism across various tissues. Compared to exercise undertaken between ZT3 and ZT4 (see Fig. 3), a single bout of treadmill running at ZT15–16 initiates a robust response to circadian muscle metabolism, including upregulation of glycolysis, FA oxidation, and oxidation of branched-chain amino acids [16, 166]. The selective transcriptional activation of CHO-related genes by early active phase exercise coincides with altered concentration of blood-borne glucose and muscle glycogen, as well as altered HIF1A activity in the skeletal muscle after exercise [16]. In this regard, a recent study reports that muscle HIF1A mediates the increased glycolytic response to forced exercise undertaken at around ZT3–4 in the early inactive phase but is dispensable for endurance performance and the function of the muscle clock [167]. Collectively, exercise at around ZT15–16 in the early active phase induces robust circadian effects on the muscle clock and metabolic responses in mice (Fig. 5).

### Liver

The liver plays an important role in regulating whole-body metabolism by preserving systemic glucose levels in response to exercise and during the post-exercise recovery period. During exercise, hepatic glucose output increases to meet the demands of working muscles and to maintain whole-body blood glucose concentrations. During prolonged (> 90 min) continuous exercise in both mice and humans, and in the face of a decline in muscle and liver glycogen levels, the provision of exogenous CHO becomes necessary to maintain the rate of muscle CHO oxidation. Upon cessation of fatiguing exercise, and with an adequate supply of CHO during recovery, muscle glycogen stores are reple­nished with glycogen depletion, providing a strong drive for their resynthesis [168].

Liver glycogen content is a robust circadian gauge that tracks the daily oscillations in CHO metabolism, exhibiting peak levels at around ZT23–1 in mice [73] (see Fig. 3). The liver clock regulates the circadian control of glycogen synthesis and glucose transport in a cell-autonomous manner via transcriptional control of rate-limiting enzymes, such as glycogen synthase 2 and hepatic glycogen-targeting PP1 regulatory subunit (GL, encoded by *Ppp1r3b*) [169–172]. These genes reach peak expression levels after the onset of the active phase. Compared to the sedentary state or early inactive phase, early active phase exercise at around ZT15–16 in mice selectively amplifies the liver–muscle connectivity and increases the abundance of exercise-induced messengers, or exerkines [5], including lactate, kynurenine, and 3-aminoisobutyrate, after exercise [166]. Together, early active phase exercise synergizes with the liver clock for liver-muscle crosstalk via CHO metabolism and exerkines in mice (Fig. 5).

### Adipose tissue

The adipose tissue is an important lipid storage depot and source of adipokine release in response to exercise. In rodents, the time of day when exercise is performed stimulates a tissue-specific lipolytic response; during the early active phase (ZT15–16), there is an exercise-induced activation of lipolysis from adipose tissue, whereas exercise undertaken at around ZT3–4 is associated with liver lipolysis [166, 173]. Adrenergic stimulation is a common trigger for adipose tissue lipolysis, and there are coordinated increases in genes related to lipolysis and mitochondrial biogenesis, including adrenergic receptor β2 *(Adrb2)* and *PGC1α,* in inguinal adipose tissue when exercise is undertaken at around ZT15–16 in the early active phase [173]. *Adrb2* expression is positively correlated with rhythmic lipolytic activity in synchronized adipocytes *in vitro* [173]. Several metabolic hormones, regulatory T-cells within adipose tissue, and the adipocyte clock collectively regulate the daily rhythm of lipolysis, which peaks at the onset of the active phase [174–176] (see Fig. 3). In line with this, early active phase exercise selectively increases serum levels of FAs and triglycerides in lean male mice [173]. Although adipokines, such as the transforming growth factor-β2 (TGFβ2) and adiponectin, mediate several health benefits of exercise [5, 177, 178], if and how the timing of exercise differentially regulates adipokine functions remains to be determined. Together, early active phase exercise at around ZT15–16 in mice promotes adipose tissue lipolysis and increases circulating levels of FAs to meet the contractile demands of muscle tissues (Fig. 5).

## Therapeutic potential of optimal timing of exercise

Given the interaction between circadian biology and exercise responses, we propose a conceptual framework to explain this circadian–exercise axis and discuss how this connection is altered by chronic metabolic disease states that are underpinned by a physically inactive lifestyle. We then examine the therapeutic potential for appropriately timed exercise to help remedy some of these conditions and shed light on potential clinical approaches.

### Targeting the circadian-exercise axis

There is growing evidence supporting the notion that the circadian clock system and external time-of-day cues have important effects on exercise biology. As such, we propose that external time-of-day cues, including but not limited to meal timing and exercise timing, influence exercise performance and many phy­siological responses to exercise via coordinated actions between circadian clocks and clock-output proteins in the skeletal muscle and other organs. For example, circadian clocks in the skeletal muscle and the liver establish synchronized daily activities in fuel storage that match the time of day for peak exercise performance in mice (see Fig. 3). Exercise training requires the muscle clock to induce transcriptional changes in multiple organs, including the skeletal muscle, white adipose tissue, liver, and heart [142, 143], indicating the essential role of the muscle clock in exercise responses. Timing of meals is sensed by the white adipose tissue via AMPK signaling and synchronizes the circadian clock and metabolism in the skeletal muscle to enhance exercise endu­rance, which suggests that inter-organ crosstalk within a daily cycle is crucial in the circadian−exercise axis [48, 49].

The circadian-exercise axis is best illustrated through circadian muscle metabolism, which represents the flexible interaction between the circadian clock and nutrient-sensing pathways. The core feedback loop of the muscle clock is aligned with light/dark cycles via the SCN. It regulates circadian CHO metabolism and myofibril assembly via clock-output proteins (e.g., MyoD1 and HIF1A). Nutrient-sensing pathways (e.g., the mechanistic target of rapamycin [mTOR], AMPK, and PPAR) couple muscle lipid meta­bolism and mitochondrial biogenesis to meal timing [179, 180]. Specific moderators, including the circadian nuclear receptors REV-ERB and ROR, integrate time-of-day signals from both the circadian clock and nutrient sensors.

The timing of exercise aligns both the circadian clock and nutrient-sensing pathways, and modulates physiological responses to exercise, at least in mice. The enhanced physiologi­cal responses from endurance exercise at the early active phase are likely because the early active phase (see Fig. 3) is the time window during which both the circadian clock and nutrient-sensing pathways are most sensitive to entrainment. As such, when combined with an extended eating window, a sedentary lifestyle uncouples circadian networks between the circadian clock and nutrient-sensing pathways, thereby exacerbating several metabolic and age-related diseases, including obesity, diabetes, and sarcopenia. This impaired circadian–exercise axis can be restored, in part or whole, by appropriate timing of exercise and meals (i.e., TRE) and presumably by timed medication. Such a hypothesis requires further study to establish cause and effect.

### Metabolic diseases

As a major risk factor for several chronic metabolic diseases, obesity is associated with profound remodeling of circadian clocks and metabolism in both mice and humans. In the skeletal muscle taken from men and women with obesity, the expression of the core clock genes, *CRY1* and *DBP*, is downregulated while indivi­duals with type 2 diabetes present with impaired circadian oscillation of the core clock genes *BMAL1*, *CLOCK*, and *PER3* in isolated skeletal muscle biopsies [181, 182]. This observation may indicate a lack of temporal synchronization with regard to muscle meta­bolism. Indeed, obesity markedly alters the rhythmic metabolome in mouse skeletal muscle and impairs the abundant rhythmic metabolite correlation between the liver and the serum, as well as that between the muscle and the serum, suggesting decreased liver–muscle circadian crosstalk via circulating cues [183]. The physiological manifestation of this asynchronous rhythmicity is that obese mice exhibit metabolic inflexibility (the inability to transition between different fuel sources to meet the prevailing energy demands and hormonal milieu) upon exercise at around ZT3–4 in the early inactive phase [184].

Nevertheless, the appropriate timing of voluntary exercise holds promise to amplify some of the metabolic benefits of exercise. Compared to voluntary exercise in the early active phase (ZT12–16; see Fig. 3), exercise in the late active phase (ZT20–24) leads to greater reductions in weight gain, improvements in meta­bolic flexibility, and enhancements in systemic insulin sensitivity in diet-induced obese mice [185, 186]. In humans, afternoon exercise training improves systemic insulin sensitivity in older men with prediabetes or type 2 diabetes [187, 188]. High-intensity interval training (HIIT) undertaken in the afternoon (4:00 p.m.) induces greater accumulation of muscle lipids, a trait associated with the paradox of the athlete [189], in men with type 2 diabetes, compared to morning HIIT (at 8:00 a.m.) [190]. In men and women with type 2 diabetes, HIIT performed in the morning is associated with higher levels of cortisol, C-reactive protein, and N-terminal pro-B-type natriuretic peptide (a marker of heart failure) than that in the afternoon [191]. While the 24-h glucose profile does not differ between morning and afternoon exercisers in that study, morning exercise increases blood glucose during the 2-h post-exercise period in both men and women, while glucose is unaltered following afternoon exercise [191]. A cohort study of 92 139 UK Biobank participants shows that individuals who engage in moderate-to-vigorous physical activity in the midday to afternoon (11:00 a.m.–5:00 p.m.) have lower risks of all-cause and cardiovascular disease mortality, compared with morning exercisers (5:00 a.m.–11:00 a.m.) [192]. While the results of these human studies are promising, it is unclear whether acute responses to a single morning or afternoon exercise bout would lead to long-term improvements in symptoms underlying metabolic diseases [193–195]. This is an area that warrants further study.

A combination of optimal exercise and meal timing has the potential to harmonize peripheral clocks and metabolism to bring about the most favorable health benefits [11, 12]. Resistance-based exercise immediately after feeding (close to early active phase resistance exercise) and night-restricted feeding collectively prevent weight gain, central obesity, and hepatic steatosis, and improve glucose tolerance in high-fat diet-fed male and female mice [196]. The combination of TRE and HIIT lowers the percentage of glycated hemoglobin A1c (a marker of glucose control over several months) and induces greater loss of total and visceral fat mass in women with overweight/obesity, compared to either TRE or HIIT alone [197]. Further studies examining the interactive effects of exercise and meal timing in a variety of clinical cohorts are urgently needed.

### Age-related diseases

Aging is associated with circadian misalignment as manifested by impaired circadian rhythmicity between tissues, including the skeletal muscle, along with a reduction in the number of rhythmic genes, indicating a loss of circadian control of physiology with aging [198]. A multimodal cell atlas of the aging human skeletal muscle reveals that circadian co-repressor genes such as *PER1* and *PER2* are decreased, whereas circadian activator genes such as *BMAL1* and *CLOCK* are increased in aged myo-nuclei [199]. Thus, aging impairs the circadian clock systemically, including the skeletal muscle.

The age-related loss of the skeletal muscle mass (i.e., sarcopenia) is devastating in aging humans [200–203], resulting in a loss of locomotor functions predisposing to impairments in activities of daily living and a loss of independence. Furthermore, sarcopenia is commonly exacerbated in individuals with overweight/obesity [204]. In the skeletal muscle, aging causes defective mitochondrial energy metabolism and protein turnover [205]. Aging and the accompanying loss of voluntary physical activity would be expected to impair circadian clocks in the musculoskeletal system. In fact, circadian clock dysfunction results in early-onset degeneration of the musculoskeletal system in mice [100, 133, 206, 207]. Similarly, Alzheimer’s disease is associated with sundowning syndrome [208], which reflects disrupted circadian connection between the brain clock and the muscle clock. While exercise is not feasible for many patients with Alzheimer’s disease, night-restricted feeding reduces brain pathology, restores sleep–wake cycles, and improves cognition in mouse models of Alzheimer’s disease [209]. Furthermore, results from a recent study suggest that a morning bout of moderate exercise (starting at 9:00 a.m.) improves working memory or executive function in older adults, which is associated with increased post-exercise increase in serum brain-derived neurotrophic factor (BDNF) [210].

In summary, preclinical studies and several human investigations show promising results regarding the appropriate timing of exercise to amplify health benefits. However, data from large-scale, long-term clinical trials in various populations (young, old, overweight/obese, etc.) are lacking. Furthermore, caution should be taken when extrapolating the findings from mouse studies to humans [31, 88, 89]. Inter-species differences (Table 1) include but are not limited to (i) scaling issues due to body size, which results in a 7-fold difference in metabolic rate and turnover [31, 88]; (ii) diet quality, composition and timing, meal size, and diurnal versus nocturnal activity patterns [17, 88, 211, 212]; (iii) fiber types wherein human myofibers shorten more slowly and lack type IIB fibers [213–215]; and (iv) patterns of substrate handling and oxidation during exercise, wherein mice have a greater reliance on extracellular substrates from metabolic tissues (i.e., blood glucose, FFAs, and lactate) while humans rely to a greater magnitude on fuels stored in the ske­letal muscle (muscle glycogen and intramuscular triglycerides) [88–90]. In the final analyses, the optimal timing of exercise depends on many variables, such as circadian phenotype, current fitness, metabolic health status, and the entrained time of waking and meals. The mode, duration, and intensity of exercise are also important variables to consider, while social and family/work responsibilities and commitments have a large bearing on any recommendation or guideline that may eventuate. These are all factors that need to be carefully considered in future cli­nical trials.

**Table 1:** Comparison of key variables of physical activity between laboratory rodents and humans.

Key variable	Laboratory rodents	Humans
Activity pattern	Nocturnal physical activity, i.e., high activity during night phase	Diurnal physical activity, i.e., high activity during day phase
Phase mapping	The SCN central clock and peripheral clocks are synchronized in phase	Unresolved. In diurnal non-human primates, all tissue clocks are inversely related to mice and flies except for the SCN clock
Fiber type	Higher proportion of type IIB and IIX fibers with abundant oxidative enzymes; Slow oxidative fibers (type 1), fast oxidative glycolytic (type 2A, 2X^a^), fast glycolytic (type 2B); Smaller fiber thickness; Faster maximum shortening velocity and tension development	Higher proportion of type I and IIA fibers with lower levels of oxidative enzymes; Slow oxidative fibers (type 1), fast oxidative glycolytic (type 2A), fast glycolytic (type 2X^a^); Larger fiber thickness; Slower maximum shortening velocity and tension development
Metabolic reliance	Greater reliance on extracellular substrates from metabolic tissues (blood glucose, FFAs, and lactate)	Greater reliance on fuels stored in skeletal muscle (muscle glycogen and intramuscular triglycerides)
Body size	Higher basal metabolic rate per unit mass; Lower maximal metabolic rate per unit mass	Lower basal metabolic rate per unit mass; Higher maximal metabolic rate per unit mass
Diet quality and composition	High carbohydrates, moderate protein, and low fat; Fixed macronutrient ratio in laboratory mice	Wide range in sugar, fat, fiber, and processed food intake; Diverse composition related to culture and personal preference
Meal timing	No structured meals; Continuous eating throughout night phase	Discrete meals (breakfast, lunch, dinner); Diurnal eating pattern

a Note that humans do not have type 2B fibers and fuel metabolism of type 2X fibers differs between laboratory rodents and humans.

## Challenges in the field

There are several unanswered questions to be addressed by future research with the twin goals of understanding the mechanistic basis underlying the circadian-exercise axis, and leveraging this knowledge to improve clinical outcomes.

### Systemic and tissue mediators

First, systemic mediators and tissue clock-output proteins of circadian–exercise biology remain largely unknown. Exerkines provide a link between exercise-induced responses and inter-organ communication, with more than 30 exerkines identified to date [5], including interleukin-6, interleukin-13, 2-hydroxylbutyrate, as well as lactate and its derivative N-lactoyl-phenylalanine (Lac-Phe) [166, 216–219]. Lactate is a critical energy metabolite, a gluconeogenic substrate, and a signaling messenger linking working muscle and other organs [86, 220–225]. Diurnal rhythms of lactate and Lac-Phe in the circulation are strongly associated with exercise responses and exercise capacity [48, 226], although the role of such rhythmicity is yet to be determined. Diurnal rhythms of exerkines in the circulation may be entrained by circadian clocks in central and peripheral tissues [227]. Some clock-output proteins including muscle PLIN5 and adipose AMPKα2 can entrain the diurnal rhythmicity of exerkines including lactate, succinate, and Lac-Phe in the circulation [48, 49]. In addition, clock-output proteins within the skeletal muscle and beyond may stimulate translational exercise research, because ultimately organ functions should be synchronized for optimal exercise responses/adaptations. The liver and the microbiome are the least studied organs in relation to their effect on the circadian-exercise axis, but are accessible for effective therapeutics, such as modified siRNAs (e.g., Inclisiran) and an *Escherichia coli* chassis [228, 229]. The translational potential of circadian-exercise mediators is immense, particularly for metabolic diseases, and more broadly, cardiopulmonary health and cognition-impaired conditions.

### Phase mapping

A deeper understanding of phase mapping between mice and humans is required before large-scale clinical studies can be undertaken (Table 1). In non-human primates, all tissue clocks are inversely related to mice and flies except for the SCN clock [38]. In humans, peripheral clocks appear to be in phase with their counterparts in mice, based on computational estimation from a large cohort of postmortem samples [41, 230–232]. However, circadian clocks in some brain regions, including the prefrontal cortex, as well as the dorsal and ventral striata, are not in phase concordance with the mouse clock system [233–235]. To address this challenge, validation of circadian biomarkers and development of point-of-care testing technology [236, 237] for human circadian clocks will be required.

### Sex-related differences

Sexual dimorphism is widely present in skeletal muscle mass, fiber composition, contractile function, and metabolism, which has a significant impact on physiological responses to exercise [215]. Males generally have larger muscle mass [213], a greater ratio of type IIA to type I skeletal muscle mass [213], and less reliance on FAs as substrates during exercise [238], compared to females [213]. For example, in vastus lateralis muscles of men, type IIA fibers have the largest cross-sectional area, whereas in females, type 1 fibers are the largest [239]. Given these differences, the skeletal muscle adapts to exercise quite differently between males and females [238, 240]. Emerging evidence indicates that females are more robust in maintaining circadian rhythms (i.e., amplitude and rhythmicity) than males [41, 49, 128, 241]. The cardiovascular fitness is associated with the timing of physical activity bouts in type 2 diabetics, which is varied by sex [242]. However, prospective studies are needed to elucidate sex-related differences in circadian physiological responses to exercise.

### Exercise modality

While much of the work to date has focused on endurance-based exercise, the modality of exercise may modulate circadian physiological responses to exercise training. Resistance- and endurance-based exercise induces differential responses in the muscle mediated by different signaling pathways [243]. Maximal force and power are higher in the late afternoon (4:00 p.m.–6:00 p.m.) compared to the rest of the day, especially the early morning (7:00 a.m.–8:30 a.m.), and this morning deficit in resistance performance can be attenuated by long-term resistance training at a specific time of day [78, 244–248]. Nevertheless, the optimal time of day for resistance training to enhance exercise responses is not conclusive, with the majority of studies favoring the early morning hours [245–247]. Mechanistically, the time-of-day effects of resistance training may rely on the coordinated actions of mTORC1 and the BMAL1–irisin axis in the skeletal muscles [244, 249]. Further, small doses of intense exercise before each main meal lead to better postprandial glycemic control than a single bout of endurance exercise [250]. Comparative human studies addressing the effects of exercise modality, circadian phenotype, meal timing, and exercise timing will move us closer to personalized exercise prescription.

## Conclusions

Accumulating evidence from both basic research and preclinical models has established the role of circadian clocks and their influence on exercise responses. This has led to an emerging model of the circadian–exercise axis as a foundation for future research. In this model, external time-of-day cues regulate exercise biology via the circadian clock system, linking circadian clocks, metabolism, and exercise. At this time, as we witness the evolving and rapidly growing field of circadian biology, it is tempting to speculate that with greater mechanistic insights underpinning circadian biology and responses to physical activity, clinicians with sufficient understanding of circadian medicine will be confident to include time-of-day exercise recommendations to improve health outcomes among their patients. Clearly, there is little danger that investigators in this field will run out of research questions in the decades to come!
